# Regular Meeting of the Massachusetts Veterinary Association

**Published:** 1890-08

**Authors:** Austin Peters

**Affiliations:** Secretary


### SOCIETY PROCEEDINGS.

*Regular Meeting of the Massachusetts Veterinary Association at 19 Boylston Place, Boston, June 25th, 1890.*—The President and Vice-Presidents being absent, Dr. W. Bryden was elected chairman for the evening.

Members present—Drs. W. Bryden, L. H. Howard, A. Marshall, W. Peterson, J. M. Skally, C. Winslow. Essayists present—Drs. Hadcock and Burr. Honorary member—Dr. J. H. Stickney.

The question of Dr. K. Winslow's resignation was considered again. After a good deal of discussion, Dr. Peterson moved that if said Dr. Winslow still be in arrears at the next meeting he be expelled, the Secretary to notify him at once. Motion seconded by Dr. Marshall. Carried.

Dr. Hadcock then read a paper upon azoturea.

GENTLEMEN: I am inclined to think that azoturea, as it appears in the stables of a horse railway company, will, as a subject, not be uninteresting to you, because it has seemed to me that there are in one way and another certain facts which should be considered as a part of the natural history of the disease which, so far as known, has never yet been so given in the books. Besides which, I have certain ideas as to the management and medical treatment of the animals, concerning which I should like to have your opinions and criticisms.

### CONDITIONS OF ITS APPEARANCE.

It is said that azoturea always follows a more or less extended period of idleness with full grain feed, and in horses having a blocky conformation; but the circumstances under which it sometimes undoubtedly occurs in my practice leads me to doubt this, as I have witnessed it in horses that have not been kept on allowances of grain feed while standing idle. In two positive instances horses (they were both mares) that had worked for a long time every day, were attacked on the road—one completely helpless in all four legs, the other in the two hind extremities only.

There are numerous instances to which horses that have been laid off from regular work, for some reason or other, have still been exercised for as much as two miles a day, then put to work, and developed the disease in the usual way.

Cases of undoubted azoturea have been shown me in horses that were absolutely thin in flesh and not of the blocky conformation—which we have been told is an absolutely necessary foundation for an attack. And it is not a particularly uncommon thing to have horses taken as if with spasmodic colic, while away from home, treated for that, apparently recover, put into their own stall, and two hours afterward fall helpless—sometimes in all four extremities.

It is a matter of conviction with me that the helpless condition is a result of tonic spasm of certain of the voluntary muscles—more particularly and ordinarily of those of the gluteal and posterior lumbar regions, but rarely the anterior part of the body or of both these together.

Paralysis, so far as this condition is understood to mean inactivity of the muscles, is not due to pressure upon some part of the cord or brain, in my opinion, but to the urea in the blood which must be eliminated from it. This office is naturally performed by the kidneys mainly. If the amount of the poison in circulation is not too great for them, the kidneys will strain it out and the animal will recover. If it is too great they will be overworked, congested or even softened, and the animal will die.

In the milder cases I give a ball of 8 or 10 drachms of aloes and keep the animal as quiet as possible with the slings loosely under him, unless he is fretted by them, and generally a good hot blanket over the lumbar region. In the more severe cases I think it best to insist more firmly upon the slings, and with them my first treatment is to put the animal on his feet and keep him there if it can possibly be done by means of any known method. The aloes is followed by the administration of sulphate of magnesia in eight ounce doses. In very severe cases the salts are repeated three times in the twenty-four hours. They may be given dissolved in water. Hot blankets wet with mustard water are packed over the kidneys, and carefully attended to. Urine must be drawn at least twice in the twenty-four hours, and if the animal is down he must be turned every six or eight hours regularly from the commencement. I also solicit an action of the bowels by the use every eight hours of an injection of soap-suds or warm water, and one ounce of glycerine. Let him have all the cold water he wants. Bran mash may be given him to eat, with very little hay to keep him quiet. Stimulants, I think, spirits of nitre, etc., do harm in the first stages, but after two or three days they seem to have a good effect. If given at first they seem to increase the uneasiness. There seems to be no danger from superpurgation. I have never been able to produce it; I have given 10 drachms of aloes and 2 pounds of sulphate magnesia with scarcely more than a laxative action of the bowels. Fresh air in currents must be supplied in abundance. If the attempt to sling at first be unsuccessful, I do not attempt it again for two or three days, the slings to be of the latest pattern, as poor slings do more harm than good.

When the horse is able to stand without the slings they should at first be placed under him at night, as there is some danger that the exertion in rising may cause rupture of the blood-vessels. This accident occurred in one case that I was interested in, causing the death of an animal that had practically recovered from the azoturea.

The results in my practice from this treatment have been very successful, as high as 90 per cent. of the cases treated as described having recovered. In this connection, however, it must be considered that we have intelligent stable foremen who are able to recognize the disease promptly, and also understand its requirements in nearly all cases. The remedies are applied early; the necessary appliances are near at hand, which, of course, helps greatly to lessen the death rate.

The following discussion ensued: Dr. Howard agreed with the essayist that a horse need not be even plethoric or lying idle in order to have azoturea, and cited an instance in his practice of a mare, then in flesh, working in a coal cart, taken while at work without having been previously laid off. Dr. Winslow said that his cases had all been of the usual character. Dr. Skally had never had a case in a mare in his practice. Dr. Peterson agreed with the essayist that horses can be taken with azoturea while working, without having any previous state of idleness. Dr. Marshall has generally seen this disease in horses in good condition after a few days rest. Dr. Marshall then asked the essayist if the atrophy of the muscles in the anterior crural region—so often seen as a result of azoturea—generally recovered. Dr. Hadcock said, in his experience, yes. Dr. Stickney said his experiences had been with horses in good flesh. He then gave an instance of a mare having symptoms simulating those of azoturea; but she was advanced in pregnancy, and finally foaled in the slings, afterwards making a good recovery. She had the same trouble in two subsequent pregnancies, and both times foaled in slings. Pregnant women often have trouble with their kidneys, and is it not possible that mares may have a similar disturbance? He also spoke of the conformation of a horse being deceitful often in judging of the condition ; an angular, loosely coupled horse may be in better flesh than a compact, round-turned one, and yet his appearance may be the reverse. Dr. Bryden then spoke of the treatment of cases of azoturea. In his opinion the medicinal treatment was of less importance than the mechanical management, such as slinging; or, if the animal was unable to rise, the turning over frequently, and general attention to his comfort. Dr. Marshall then moved that the Association tender Dr. Hadcock a vote of thanks for his paper, and that he be elected a member. Seconded by Dr. Winslow and carried unanimously.

Dr. Alexander Burr then read an account of his experiences as meat inspector for the Boston Board of Health at the Brighton Abattoir.

Mr. President and Gentlemen of the Massachusetts Veterinary Association :

Permit me to present to you a short account of my experiences as inspector at the Brighton Abattoir for the Board of Health of the city of Boston since October 1st to April 1st.

In passing, I may say that the Abattoir Company is a corporation who rent sections of their premises to the butchers of Boston for slaughtering purposes. Previous to my appointment they had been accustomed to a simple mode of inspection, which consisted principally in an examination of the dressed meats. They were, therefore, unprepared for a careful professional examination of the live animals, followed by further inspection during the process of dressing.

The course I have pursued has been to make a tour of the different yards every morning, to gain an idea of the character of the animals, marking such as look suspicious so that they could be identified when dead. At first, I was regarded by the butchers with more or less, perhaps natural suspicion, especially when the carcass and viscera showed that anything was abnormal, and a desire was shown on my part that they should remain together. In justice to the butchers, it must be said that now they do not intentionally buy diseased animals, or handle unclean or unwholesome flesh ; but having them they desire to lose as little as is possible.

The rest of my time has been spent in walking through the different houses, observing the animals while being dressed, and afterward, should any part of the viscera be accidentally or intentionally disposed of, still another examination is made of the offal, when, should anything of a diseased character be found, the carcass to which it belongs is immediately hunted up. I have sometimes found in the rendering house, lungs in an advanced stage of chronic tuberculosis, with abscesses, cheesy matter, grapes, etc. It then becomes my duty to find the animal from which they came. Having examined every carcass, and found one with a number of small grapes (tuberculous nodules) on the costal and diaphragmatic pleuræ, which had not been removed, one should feel justified in condemning such, even if it looked and weighed well.

During the above time there have been killed 15,506 cattle, calves and sheep, among which there have been 880 cows. Of the above, there have been condemned 10 cattle, 31 calves, and 5 sheep. Of course, the above does not include animals arriving dead, of which sheep amount to a large number.

Among the above condemned animals (that of the larger ones) have been found the following diseases:

Tuberculosis, Perinephritic abscess, Pylo-phlebitis, Anthrax and Texas fever.

In addition to these, I shall also describe a few cases of actinomycosis, of which there have been found seven. This lesion, as was at one time thought, is not confined to the facial region. Of the three cases with lesions found elsewhere than in the face, one was in the tongue, and two in the lungs, one of which was confined to the tongue alone.

(*a*) That having lesions in the tongue. On running one's fingers down the lateral surfaces of the tongue it felt distinctly nodular, though not apparent to the eye; on cutting into these nodules they were found to be made of small centres of yellow granular matter, covered with fibrous tissue, and the characteristic radiating fungoid growth was fully demonstrated in the usual way.

(*b*) This case presented a small tumor of left superior maxilla, consisting of small points filled with yellow matter; an examination of the lungs was made, and found the left to contain numerous large abscesses, confined to one lobe, giving it a honeycomb appearance, the spaces being filled with yellowish puriform matter. This case was interesting from the fact that it might have been taken for a case of tuberculosis where it has gone on to the formation of large abscesses, and it would be sure to mislead one not competent to make a microscopic examination.

(*c*) No facial lesions were found in this case, but in one lung there was a nodule made up mostly of fat, in centre of which was a calcified mass of fungoid growth ; it had a contracted appearance and was, in all probability, an arrested stage of the disease, in which calcification had set in. This, perhaps, may be looked upon as being an unusual result of the disease under consideration, but in this instance, at any rate, the diagnosis is as certain as this form of termination is rare.

PYLO-PHLEBITIS is quite a frequent trouble, all but two cases consisting of a few small abscesses, in which case simply the liver has been condemned. The following is a description of two cases :

(*a*) The liver contained a large abscess, which had worked its way forward through the diaphragm and into the right lung, resembling a thick rubber tube, having a large abscess on either end, dumb bell shape, the spheres of which were lodged in the lung on one end, and the liver on the other. Microscopic examination of the pus showed nothing but simply micrococci.

(*b*) This case was what might be called general pylo-phlebitis. The liver was much enlarged, weighing twenty-six pounds, and one mass of miliary abscesses ; rest of organs all normal ; animal was quite emaciated, weighing only four hundred and thirty pounds. The above was condemned.

TEXAS FEVER.—Three cases of this disease arrived on the grounds, two of which were dead and at once sent to the dead house.

The third case was that of an animal which had been shot at the Watertown stock-yards, and carted into the slaughter house to be dressed ; there was a question in my mind whether the animal was not dead, or almost so, before shooting. On cutting down the median line of the abdomen, the omentum covering the rumen was exposed, and was of a dirty gray color, and smelling badly, arousing my suspicions at once. The liver was secure, which was enlarged and yellowish ; spleen appeared normal. Feeling justified in my diagnosis the animal was condemned and ordered to be at once sent to the dead house. The small number of cases of this disease is probably to be accounted for from the fact, the season being that in which Texas fever is not prevalent.

ANTHRAX.—Five cases of this most dreaded disease arrived on the grounds, three of which died, and two having been shot at the stock yards and brought here with the intention of dressing for meat. The first of these I will describe. An order came for the abattoir ambulance to go to Watertown for a steer which had been shot; it was ordered to be sent into one of the houses.

I reached the house as they were cutting down through the median line of abdomen, exposing the omentum to view, which was almost black in color and had a very disagreeable gaseous odor, putrefaction having already set in, although the animal was supposed to have been dead less than half an hour. My suspicions were at once aroused. From the rapid decomposition and my having had three cases two weeks previously, I was somewhat on my guard for anthrax, and after further convincing myself I ordered the animal sent to the dead house; the floors were then cleaned and necessary precautions taken before dressing any more on the same spot. On post-mortem examination, no marked lesions of an anthracoid nature were found, although in the form which I take to have been apoplectic, one would not expect to find any marked lesions. However, feeling sure of my diagnosis, scrapings were taken from the spleen and examined microscopically for the bacillus anthracis, with positive results, the rods having begun to form spores from exposure to the air; this left no doubt as to the diagnosis. This carcass was ordered along with hide to be at once placed in a closed rendering tank and subjected to a prolonged cooking under a pressure of fifty pounds of steam, which means a temperature of about 300° F.

TUBERCULOSIS.—The detection and detention of such cases as would seem unfit for consumption, have been a part of my work in which I have taken much interest, and rigidly examined all cows slaughtered here, as the disease is most prevalent among them. Up to date, April 1st, twenty-nine cases have been discovered, showing lesions all the way from a mere nodule to the most advanced stage. Twenty-eight of the above number were cows, the twenty-ninth being a Northern ox; of these, five only have been condemned; this means that these five had considerable thoracic and in some cases abdominal lesions.

On account of the differences of opinion as to the danger of contracting the disease from eating the flesh of those killed during its early stage, and where they have shown only trifling lesions, I have condemned only such as seem to be the worst. Perhaps it had better be said right here that the surrounding towns have no system of inspection.

Although my examinations show a considerable percentage of tuberculosis among our Eastern cows coming here, I am convinced that the disease does not prevail to anything like the extent that some of our members have reported, and possibly honestly suspect. Below are a few statistics gathered at the abattoir:

There have arrived 880 cows, of which 70 were Western, leaving 810 Eastern. Of these 880, twenty-eight were found to have lesions of tuberculosis; of the 70 Western cows not one case was found. The percentage will read as follows:

| | |
|---|---|
| Number of animals (cows and steers) killed .... | 15,506 |
| Percentage of tuberculosis ...................... | .17 |
| Of the 880 cows, Eastern and Western ............ | 3.30 |
| Of the 810 cows, Eastern only .................. | 3 60 |
| Of the 70 cows, Western only .................... | 0.00 |

Of the twenty-nine cases discovered, there was not one among them but what showed some pulmonary lesion. I do not wish to be understood as thinking there is no such thing as localized tuberculosis. This has been demonstrated by inoculation, but from my experience it would seem that invasion most frequently takes place through the respiratory passage.

Of course, we must take into consideration that the cows coming here are generally thought to be sound; that is, we do not get all the animals used in the cheaper grades of beef. Thus it will be seen that the above statistics are not the actual statistics of the State; still, I think a fair average of abattoir statistics. An acquaintance with the subject of inspection, as reported in the current professional journals of the day, will convince any one unprejudiced that we are better off than any European country reported. That is, the percentage of tuberculosis among our animals is less than in any European country. The number of animals killed outside the abattoir can only be a very small number compared with the other, and it would be unfair to think that all such are diseased, or even one-fourth of them.

So far as can be judged from my short period of inspection, even among our Eastern cattle tuberculosis exists to a much less extent than among animals in the populous centres of most European countries; and among our Western bullocks tuberculosis has almost no existence whatever, and this class of animals represent two-thirds of our cattle population.

I may add in connection with the foregoing, that in relation with the abattoir we have an establishment where fertilizers are manufactured, and dead animals of all kinds received, such as horses and cattle, many of which are cows; these animals represent a fair average of the cows of our neighborhood; having died, the owners have seldom any disposition to hide them. I have examined all the cattle brought here and so far my record is as follows:

| | |
|---|---|
| Received dead cows at abattoir from Oct. 1st, 1889, till April 1st, 1889 | 80 |
| Number found with tuberculous lesions | 6 |
| Percentage | 7.5 |

No better opportunity, it seems to me, could be found to reach a fair average of the extent to which the disease prevails among our animals.

The following discussion ensued: Dr. Howard stated that his personal experience with cattle was very limited, but hoped that Dr. Burr was right in his small estimation of the amount of bovine tuberculosis in the locality; was afraid, however, that it existed to a greater extent than the essayist judged it to, from what some of our other practitioners say, in whom he has every reason to feel confidence. Dr. Peterson thinks that a good many animals that are tuberculous are not sent to the abattoir; doubted if 50 per cent. of the creatures with the disease were sent to the abattoir. He then told of a slaughterhouse out in the country, not a great way from Boston, which he happened to visit one day, and where he saw "strange sights." Dr. Marshall said he thought there was less tuberculosis around Eastern Massachusetts than many of our members would have us believe. Dr. Stickney said he had but little cow practice, but had seen a good deal of bovine tuberculosis. He thought that Dr. Burr's statistics were not very valuable towards showing the prevalence of the disease around here, as the beef he inspects comes chiefly from the West. Dr. Burr's statistics are only correct as far as the animals brought to the Brighton abattoir are concerned, but do not prove a great deal beyond that. It is not to be wondered at that tuberculosis should exist in many of our well-bred dairy herds, as it has been carefully propagated there for years. Dr. Marshall then moved that the essayist be given a vote of thanks for his paper, and be elected a member of this Association. Seconded by Dr. Howard and carried unanimously.

Meeting then adjourned. AUSTIN PETERS, *Secretary*.

